# Toward Multitasking Pharmacological COX-Targeting Agents: Non-Steroidal Anti-Inflammatory Prodrugs with Antiproliferative Effects

**DOI:** 10.3390/molecules26133940

**Published:** 2021-06-28

**Authors:** Fedora Grande, Francesca Giordano, Maria Antonietta Occhiuzzi, Carmine Rocca, Giuseppina Ioele, Michele De Luca, Gaetano Ragno, Maria Luisa Panno, Bruno Rizzuti, Antonio Garofalo

**Affiliations:** 1 Department of Pharmacy, Health and Nutritional Sciences, University of Calabria, Ampl. Polifunzionale, Via P. Bucci, 87036 Rende, Italy; francesca.giordano@unical.it (F.G.); mariaantonietta.occhiuzzi@unical.it (M.A.O.); giuseppina.ioele@unical.it (G.I.); michele.deluca@unical.it (M.D.L.); gaetano.ragno@unical.it (G.R.); mluisa.panno@unical.it (M.L.P.); 2 Department of Biology, Ecology and Earth Sciences, University of Calabria, Via P. Bucci, 87036 Rende, Italy; carmine.rocca@unical.it; 3 CNR-NANOTEC, SS Rende (CS), Department of Physics, University of Calabria, Via P. Bucci, 87036 Rende, Italy; bruno.rizzuti@cnr.it; 4 Institute of Biocomputation and Physics of Complex Systems (BIFI), Joint Units IQFR-CSIC-BIFI, and GBsC-CSIC-BIFI, University of Zaragoza, 50018 Zaragoza, Spain

**Keywords:** COX-1, COX-2, nabumetone, molecular docking, anti-enzymatic assays, cell toxicity

## Abstract

The antitumor activity of certain anti-inflammatory drugs is often attributed to an indirect effect based on the inhibition of COX enzymes. In the case of anti-inflammatory prodrugs, this property could be attributed to the parent molecules with mechanism other than COX inhibition, particularly through formulations capable of slowing down their metabolic conversion. In this work, a pilot docking study aimed at comparing the interaction of two prodrugs, nabumetone (NB) and its tricyclic analog 7-methoxy-2,3-dihydro-1*H*-cyclopenta[*b*]naphthalen-1-one (MC), and their common active metabolite 6-methoxy-2-naphthylacetic acid (MNA) with the COX binding site, was carried out. Cytotoxicity, cytofluorimetry, and protein expression assays on prodrugs were also performed to assess their potential as antiproliferative agents that could help hypothesize an effective use as anticancer therapeutics. Encouraging results suggest that the studied compounds could act not only as precursors of the anti-inflammatory metabolite, but also as direct antiproliferative agents.

## 1. Introduction

The correlations between inflammation and cancer onset and progression are in many cases proven by increasingly clear evidence. The transformation of a tissue from inflamed to cancerous has often been observed in the case of gastric, colorectal, and prostate cancer. High levels of prostaglandins (PGs), recognized as mediators of inflammation, have been detected in breast, head, neck, lung, and colon cancers [1,2,3,4,5,6]. It is well known that excess of PGs derives either from an increase in the level of the precursor arachidonic acid, coming from injured cell membranes, or from an over-expression of cyclooxygenase (COX), the enzyme that promotes the conversion of the acid into mediators of pain amplifying inflammation symptoms. At least two isoforms (COX-1 and COX-2) of the three identified for this enzyme have been postulated to participate in this mechanism [7,8]. In particular, COX-1 is expressed in several tissues already under physiological conditions, whereas the expression of COX-2 seems to be induced mostly after pathologies by growth factors, tumor promoters, etc. [9]. COXs then represent major targets for non-steroidal anti-inflammatory drugs (NSAIDs), among the most prescribed therapeutics for the treatment of inflammatory diseases. However, prolonged use of NSAIDs is associated with gastro-toxicity and renal damage, as they non-selectively inhibit both the constitutive COX-1 and the inducible COX-2. On the other hand, selective COX-2 inhibitors do not show gastric side-effects, though they have been found to be cardio-toxic, especially after long-term use [10]. Although both COX isoforms are over-expressed in many forms of cancer, the crucial role of COX-2 has been proven during cancer progression and invasiveness [11,12].

Accordingly, several NSAIDs were demonstrated to possess an inhibitory effect on carcinogenesis and cancer spreading. For example, selective COX-2 inhibitors showed suppressive activity against azoxymethane-induced aberrant crypt foci in rat colon, by a mechanism not yet fully understood, but probably connected with anti-inflammatory action [13,14,15]. The use of NSAIDs has recently been associated with a reduced risk of skin cancer, although such a result needs to be conclusively proven [16]. In addition, the use of COX-2 targeting agents may help limit the production of prostacyclines, reflected in the inhibition of platelet aggregation, which has been shown to promote the growth and metastasis of cancer cells [17,18,19,20].

The pro-tumorigenic effect attributed to COX-2 seems to be due to different mechanisms, including the induction of cell proliferation, apoptosis inhibition, and the host’s immune response suppression. COX-2 has also been considered as a promoter of vascular endothelial growth factors (VEGF) production, which would favor angiogenesis. Thus, selective COX-2 inhibitors represent promising therapeutics against cancer [21]. Moreover, high levels of reactive oxygen species (ROS) leading to aberrant redox signaling have been observed in many forms of cancer. This would be reflected in a role of chronic inflammation characterized by abnormal levels of ROS during cancer onset [22,23].

The anticancer activity of NSAIDs is also attributed to further alternative mechanisms including down-regulation of survivin expression, inhibition of the peroxisome proliferator-activated receptor γ (PPARγ), inhibition of the nuclear factor kappa light chain enhancer of activated B-cells (NFkB), and induction of the NSAID-activated gene (NAG-1) expression. All these factors play crucial roles in apoptosis modulation, thus in cancer emergence, confirming a potential role of NSAIDs in cancer treatment [24,25].

**Figure 1:**
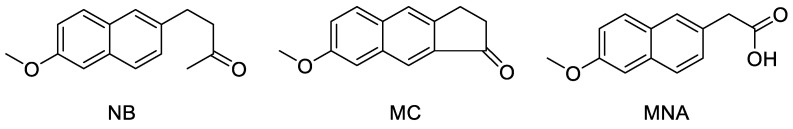
Chemical structure of the three studied compounds NB, MC, and MNA.

In an early study, nabumetone (NB), a prodrug generating a rather selective COX-2 inhibitory activity in vivo, showed a chemopreventive effect on mammary carcinogenesis induced by *N*-methyl-*N*-nitrosourea, probably suppressing cell proliferation or by reducing the number of malignant cells via apoptosis [26]. However, no proof has been provided in order to establish whether this property was attributable to NB itself or to its metabolite 6-methoxy-2-naphthylacetic acid (MNA) endowed with a strong anti-enzymatic activity against COX-2 (Figure 1). This does not exclude that oral prodrugs, usually thought as devoid of any intrinsic activity against a specific target, could directly act as such with alternative mechanisms. In an independent study based on the same experiments, sulindac sulfone, a metabolite of the prodrug sulindac, although inactive against COX, showed a dose-dependent inhibitory activity on carcinogenesis [27]. In this case, it was therefore possible to exclude that the antitumor activity was mediated by the enzyme inhibition. Finally, NS-398, a selective COX-2 inhibitor structurally related to nimesulide, was found to possess antiproliferative activity against colorectal cancer cell lines [7]. The latter result was recently confirmed when the overexpression of COX-2, together with higher levels of VEGF, was indicated to be able to promote endothelial lumens formation and neovascularization, therefore favoring cancer cells growth and metastasis. This effect could be counteracted by COX-2 targeting agents, which would inhibit the enzyme production [28,29,30,31]. It can be concluded that NSAIDs, although in general do not possess any intrinsic antitumor activity, may however contribute to slow down cancer onset and progression due to their peculiar anti-enzymatic mode of action.

Nabumetone is a non-acidic prodrug capable of generating in vivo MNA, its active metabolite with a preferential inhibitory effect for COX-2 rather than COX-1 [32]. After oral administration, the efficacy attributed to this drug was comparable to that of the most potent NSAIDs, along with a concomitant favorable toxicity profile. It has been proposed that the conversion of NB into MNA in the liver occurs by a steady mechanism mediated by the oxidative catalysis of CYP1A2, allowing a half-life of 24 h [33].

The majority of the studies performed on NB refer to the activity of MNA, whereas the parent ketone was not taken into consideration for the evaluation of any alternative biological activities. Although the metabolic process rate helps to prolong the anti-inflammatory effect, it is reasonable to assume that only a residual amount of the unmodified prodrug still circulates until its conversion is complete. The use of NB has been also proposed for topical application, often by a controlled absorption favored by opportunely designed formulations [34,35,36]. In fact, while the lipophilic nature of NB should allow sufficient epidermal crossing and tissues infiltration, only the inclusion of the drug molecules into special carriers, for example microemulgel systems, would guarantee almost complete skin absorption [35]. A further opportunity for the prodrug to persist somewhat longer into blood circulation would be represented by depot injection formulations. Besides the use against inflammation symptoms, an anticancer potential of NB was also envisaged [16]. 

Recently, 7-methoxy-2,3-dihydro-1*H*-cyclopenta[*b*]naphthalen-1-one (MC, Figure 1), a tricyclic analog of NB that would act with a similar mechanism, conceived to maintain comparable physico-chemical properties and share a similar metabolic activation, was synthesized to be analogously included into innovative formulations [34]. Accordingly, we found it interesting to perform a preliminary test of the antiproliferative potential of NB and MC that, although conceived as anti-inflammatory prodrugs, might be endowed with additional biological properties, i.e., anticancer activity. This way, a dual mechanism for a possible application of the drug during cancer prevention and therapy may be hypothesized, providing that the metabolic process could be controlled. Such mechanism would consist of a direct antiproliferative action exerted by the parent molecule, followed by an indirect anticancer effect due to COX-2 inhibition from the active metabolite. On the other hand, a longer half-life of such prodrugs could even result in an anti-inflammatory effect from their direct interaction with the enzyme. Furthermore, the role of COX-1 in cancer has been recently reconsidered. In fact, although this isoform was considered as constitutive and then involved in physiological processes, increased levels of COX-1 were occasionally reported in different types of cancer, particularly during the early phases of the disease [37].

Accordingly, our study started with a preliminary in silico assay based on the evaluation of the interaction of the molecules (NB, MC, and MNA) with the three-dimensional structure of the COXs active site. The results obtained suggest a possible activity for all the three derivatives, and the evidence of the higher MNA anti-inflammatory activity would be an obvious consequence of the pharmacokinetic transformations. In order to ascertain their potential as anticancer agents, the two prodrugs NB and MC as well as the metabolite MNA were investigated in vitro in two breast cancer models, by cytotoxicity assays, flow cytometry, and immunoblotting experiments. Moreover, the studied compounds were tested in an anti-enzymatic assay against both COX isoforms. Due to a preferential activity on COX-2 and a lack of toxicity on healthy cells, both prodrugs could represent starting points for the development of alternative anticancer agents.

## 2. Results and Discussion

### 2.1. Molecular Docking

**Figure 2:**
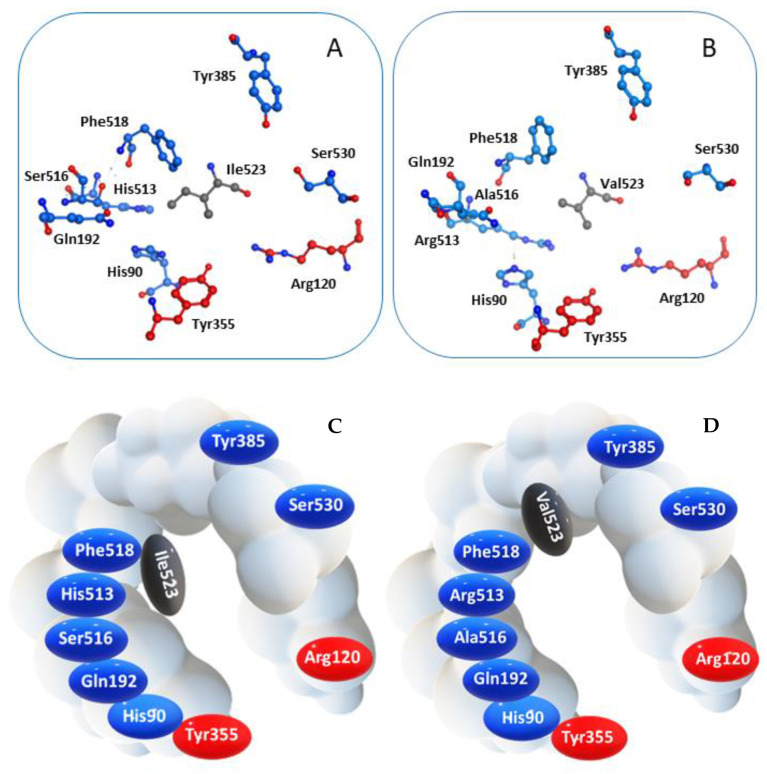
Spatial arrangement of key residues in the binding pocket of (**A**) COX-1 and (**B**) COX-2, and corresponding schematic representation highlighting the different wideness of the catalytic cavity of the two enzyme isoforms (**C**) COX-1 and (**D**) COX-2.

COX-2 is a homodimer with a half-site reactivity, because only one monomer is active at a given time [38]. The active site consists in a 22-amino acids hydrophobic pocket encompassing protein regions starting from Arg120 and ending with Tyr355, two residues that together limit the width of the pocket channel access (Figure 2) [39]. The presence of a valine residue in this enzyme, replacing the isoleucine at position 523 of the isoform COX-1, confers a wider opening of the access channel and an overall larger pocket size, allowing bulkier ligands to accommodate into the active site [40].

Molecules able to enter this region could prevent the interaction between COX-2 and its endogenous ligand, namely arachidonic acid, thus inhibiting the enzyme function. Furthermore, crystallographic data prove that Arg120 is crucial for the interaction with the carboxylic group characterizing either the endogenous substrate and several classical NSAIDs, even though non-acidic agents can still enter the pocket [41,42].

**Figure 3:**
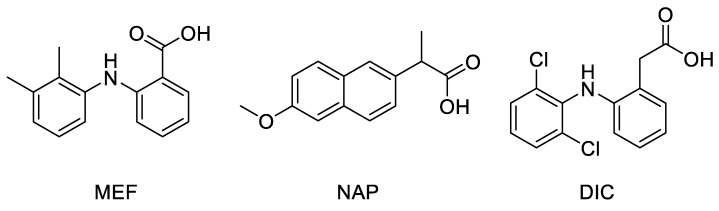
Chemical structure of crystallographic ligands MEF, NAP, and DIC complexed with COX-2 enzyme (PDB code 5IKR, 3NT1, and 1PXX, respectively).

Docking studies were performed on a crystallographic structure of COX-2 obtained from the Protein Data Bank (PDB code: 5IKR [43]), to assess the quality of molecular interactions between the active site of the target protein and compounds MNA, NB, and MC. In 5IKR, the protein is complexed with mefenamic acid (MEF), a previously identified COX-2 selective inhibitor (Figure 3). This ligand mainly interacts with Tyr385 and Ser530 at the top of the pocket, forming two hydrogen bonds through its carboxylate group (deprotonated due to its low pK value) with the phenolic oxygen of the former and the hydroxyl oxygen of the latter. Additional van der Waals interactions stabilize the complex.

**Figure 4:**
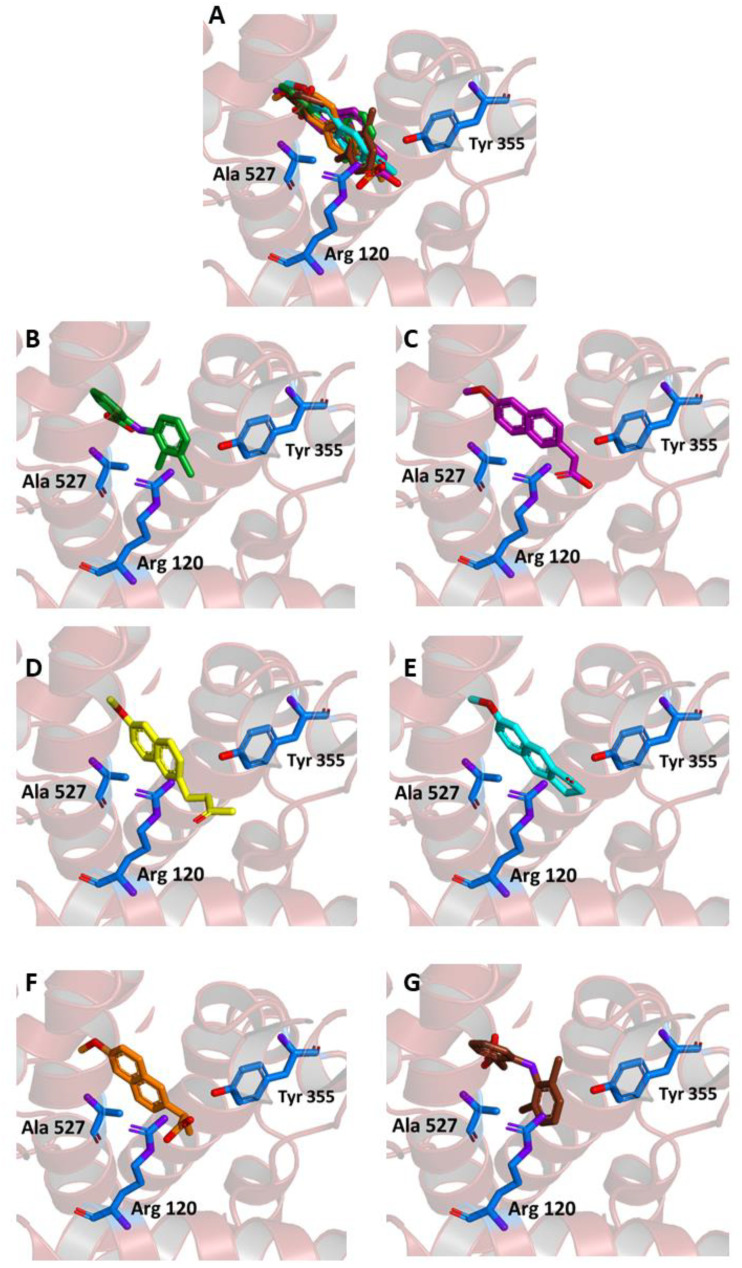
Ligand binding into the active site of COX-2. Protein backbone is represented in the background as a ribbon and only key residues are highlighted. (**A**) Superimposed binding modes of the MEF (green), MNA (purple), NB (yellow), MC (cyan), NAP (orange), and DIC (brown). The ligands are also shown separately: (**B**) MEF, (**C**) MNA, (**D**) NB, (**E**) MC, (**F**) NAP, and (**G**) DIC.

As shown in Figure 4A–E, in our experiments, the three molecules share in all cases the same position occupied by MEF in the crystallographic complex. Furthermore, all of them are able to interact through key residues of the active site. In particular, MNA binds through its carboxylate anion, the amine group of Arg120 and hydroxyl of Tyr355, by two hydrogen bonds with a donor-acceptor distance of 2.88 and 3.08 Å, respectively. NB binds through the carbonyl oxygen Arg120 by means of a hydrogen bond with a donor-acceptor distance of 2.53 Å, whereas MC forms two hydrogen bonds through its carbonyl oxygen with Arg120 and Tyr355 with a distance of 2.82 and 2.64 Å, respectively. Beside these hydrogen bonds, additional contacts between the ligands and the residues lining the enzymatic channel create hydrophobic interactions. In particular, the naphthalene moiety of the compounds establishes a π-interaction with Ala527, with an average distance of 3.80 Å.

Binding energies of −7.6, −7.6, and −8.3 kcal/mol were observed for the interaction of COX-2 with MNA, NB, and MC, respectively, indicating a favorable affinity of the three molecules for the protein. These values are comparable to the one obtained after re-docking of MEF into the crystallographic position (−8.4 kcal/mol).

To further assess the reliability of our docking simulations, we also tested the binding to our target of naproxen (NAP) and diclofenac (DIC), two NSAIDs largely used in the clinical practice. The PDB reports the crystallographic structure of the complexes of both NAP and DIC (code 3NT1 [41] and 1PXX [42], respectively) complexed with COX-2 from *Mus musculus*, which has a high sequence similarity (about 90%) with the one from *Homo sapiens*. Re-docking experiments indicated that the binding energy was −8.1 and −6.9 kcal/mol for the NAP-3NT1 and DIC-1PXX complex, respectively.

When the two drugs were blindly docked to our *Homo sapiens* target (PDB code: 5IKR), they both anchored to the expected binding site (Figure 4F,G). NAP formed two hydrogen bonds between the carboxylate anion and the amine group of Arg120, and an additional bond with the hydroxyl group of Tyr355, with a donor-acceptor distance of 2.90, 2.96, and 2.90 Å, respectively. On the other hand, DIC interacted with the protein by forming a hydrogen bond with Tyr385 with a distance of 3.16 Å (Figure 4G). The predicted affinity was −8.6 kcal/mol for NAP and −7.8 kcal/mol for DIC. These values are slightly more favorable compared to the ones obtained for the same ligands bound to the mouse protein and, more interestingly comparable to the ones obtained with our ligands (MNA, NB, and MC had affinities in the range between −7.6 and −8.3 kcal/mol, as reported above) bound to the human one.

Another metric to evaluate the similarity between the crystallographic ligand bound to COX-2 and the docking poses obtained in our simulation is the distance observed between geometric centers of selected regions of these ligands. Thus, as reported in Table 1, we calculated the distance between (i) all non-hydrogen atoms and (ii) the closest benzene ring for MEF with respect to NB, MNA, MC, NAP, and DIC. The results show that the poses obtained through our blind docking experiments were within 2 Å from the crystallographic ligand for both MNA and MC, whereas for NB, the distance was slightly larger due to the extended conformation of its –CH_2_–CH_2_–CO–CH_3_ tail. However, when the distance was evaluated by considering the closest aromatic ring common among all these ligands (Figure 4), the results were in all cases below the standard C–C distance in such a ring (1.40 Å), indicating that these structures were so close that they could be considered essentially superimposed. A small distance with respect to the geometric center of MEF bound to COX-2 was obtained for NAP and DIC, in particular for the latter, due to its closer structural similarity with MEF.

**Figure 5:**
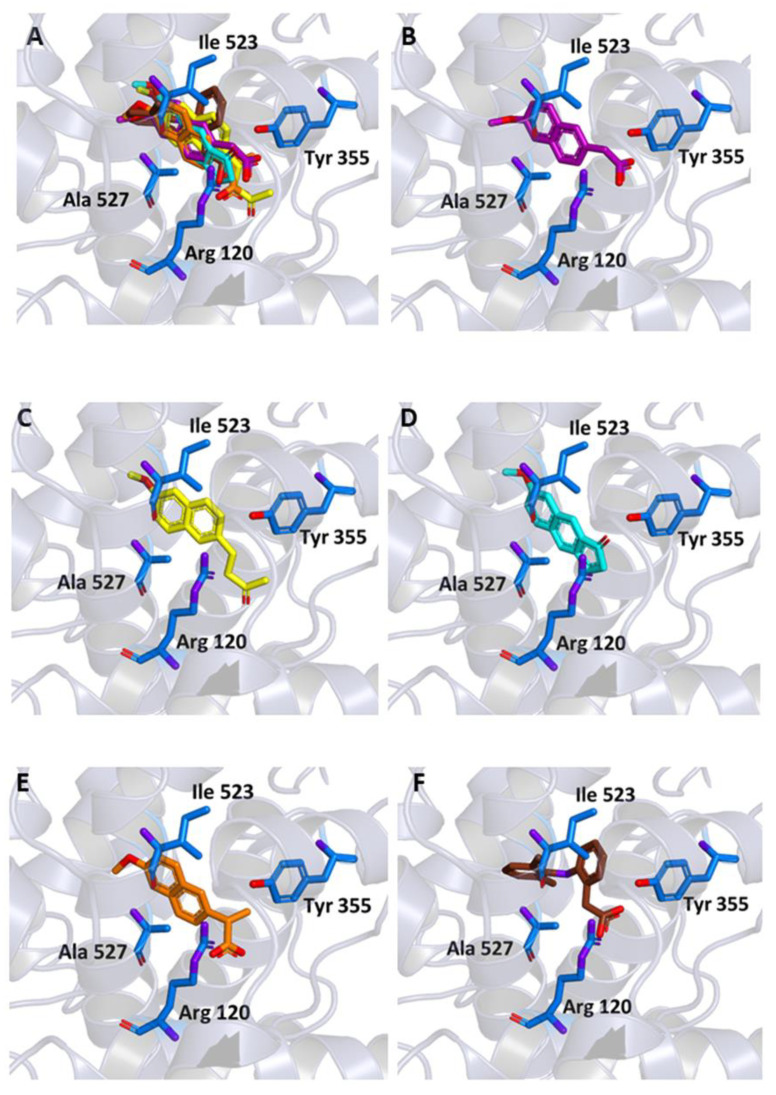
Ligand binding into the active site of COX-1. Protein backbone is represented in background as ribbons and only key residues are highlighted. (**A**) Superimposed binding modes of MNA (purple), NB (yellow), MC (cyan), NAP (orange), and DIC (brown). The ligands are also shown separately: (**B**) MNA, (**C**) NB, (**D**) MC, (**E**) NAP, and (**F**) DIC.

Similar docking experiments were carried out on a crystallographic structure of COX-1 (PDB code 6Y3C), to assess the binding of the ligands also to this target. The binding modes of MNA, NB, and MC compared to those of NAP and DIC are represented in Figure 5. MNA, NB, and MC were able to interact with key residues of the protein active site although with binding energy values less favorable than those observed for COX-2 (−6.6, −6.8, and −6.6 kcal/mol, respectively).

In conclusion, our docking data suggest a significant affinity towards both COXs for MNA, which carries an overall negative charge, and for both NB and MC neutral prodrugs. Although for classic acid NSAIDs, the activity seems related to the presence of the carboxyl group, our two compounds can as well interact with the enzyme catalytic pocket, likewise the majority of COX-2 selective inhibitors which lack such a feature [44]. These considerations do not exclude an intrinsic anti-inflammatory property or alternative biological activities for the tested compounds.

### 2.2. Biological Assays

As previously discussed, a possible future therapeutic option for certain types of cancer could reside in a dual approach based on the use of drugs possessing specific direct antiproliferative action with coadjuvant anti-inflammatory activity. Such a result could be pursued by the use of molecules possessing a given intrinsic anticancer activity and gaining the anti-inflammatory effect after metabolic transformation. NB and MC could represent first candidates due to the anti-inflammatory action of their common metabolite MNA and their direct anticancer activity. Accordingly, we tested both compounds in cell culture viability as well as immunoblotting assays on hormone sensitive and insensitive breast cancer models.

### 2.3. NB and MC Inhibit Breast Cancer Cell Survival

**Figure 6:**
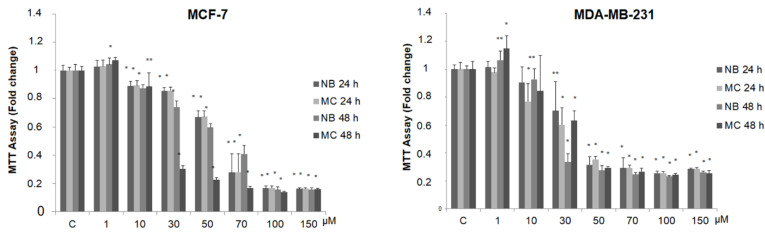
MTT growth assays in MCF-7 and MDA-MB-231 cells treated or not with increasing doses (1, 10, 30, 50, 70, 100, 150 µM) of NB and MC for 24 and 48 h. Cell proliferation is expressed as fold change ± S.D. relative to vehicle-treated cells and is representative of three different experiments, each performed in triplicate. Statistical significance was considered at * *p* < 0.05; ** *p* < 0.005. Statistical comparisons were drawn between groups by using a two-sample two-tailed *t*-test. C = control.

In order to detect any antitumor activity, we tested the effect of NB and MC on breast cancer cell viability, using two stabilized cell lines as experimental models: MCF-7 (estrogen positive, poorly invasive, and low metastasizing) and MDA-MB-231 (triple negative, highly invasive, and metastatic). For this purpose, the compounds were tested at increasing concentrations (from 1 to 150 µM) by MTT assay. As shown in Figure 6, the treatment with the two compounds after 24 and 48 h reduces cell survival in a dose-dependent manner in both cell lines. 

**Figure 7:**
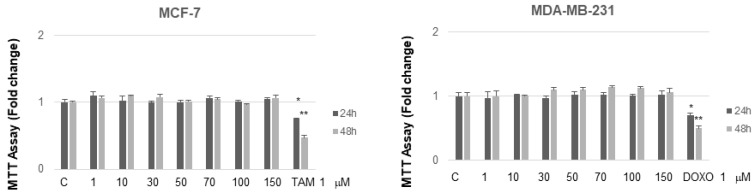
MTT growth assays in MCF-7 and MDA-MB-231 cells treated or not treated with increasing doses (1, 10, 50, 70, 100, 150 µM) of MNA and with 1 µM of either TAM or DOXO, respectively, for 24 and 48 h. Cell proliferation is expressed as fold change ± S.D. relative to vehicle-treated cells and is representative of three different experiments each performed in triplicate. Statistical significance was considered at * *p* ≤ 0.05; ** *p* ≤ 0.01. Statistical comparison was drawn between groups using a two-sample two-tailed *t*-test. C = control.

It is interesting to note that, at concentrations from 30 to 70 µM after 24 h of incubation, MC and NB were both active against MCF-7 cells and, to a major extent, against MDA-MB-231 cells. MNA was tested at the same increasing concentrations used for the other two compounds, in both cancer cell lines. As shown in Figure 7, this compound did not exert any toxic effect on MCF-7 and MDA-MB-231 compared to control. In the same assay, two known anticancer agents, tamoxifen (TAM) and doxorubicin (DOXO), reported as effective inhibitors of cell proliferation for MCF-7 and MDA-MB-231, respectively, were tested for comparison. As expected, both of these agents proved to be active at concentrations lower than 1 µM.

The half-maximal inhibitory concentration (IC_50_) values for NB and MC after 24 h of incubation are shown in Table 2.

**Figure 8:**
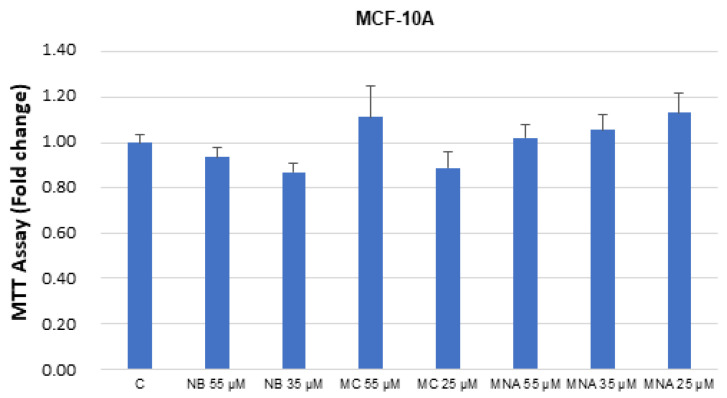
MTT growth assays in MCF-10A cells untreated (control, C) or treated with doses of NB, MC, and MNA at IC_50_ for 24 h. Cell proliferation is expressed as fold change ± S.D. relative to vehicle-treated cells and is representative of three different experiments, each performed in triplicate. Statistical comparison was drawn between groups using a two-sample two-tailed t-test.

The studied compounds were also tested in breast healthy cells MCF-10. No significant toxicity was detected up to the IC_50_ values determined in the cancer cell assays (Figure 8).

In conclusion, our experiments showed that the two studied prodrugs possess an antagonistic effect on the proliferative kinetics of cancer cell at the concentrations used throughout the experiment, even though weaker than that associated to the reference anticancer drugs. On the other hand, the anti-inflammatory metabolite MNA did not show significant activity under the same conditions. These results would suggest that the antiproliferative activity of the two prodrugs, exerted against cancerous cells only, is independent of their metabolic transformation.

### 2.4. Effects of NB, MC, and MNA on COX-1 and COX-2 Activity in MCF-7 and MDA-MB-231 Cells

**Figure 9:**
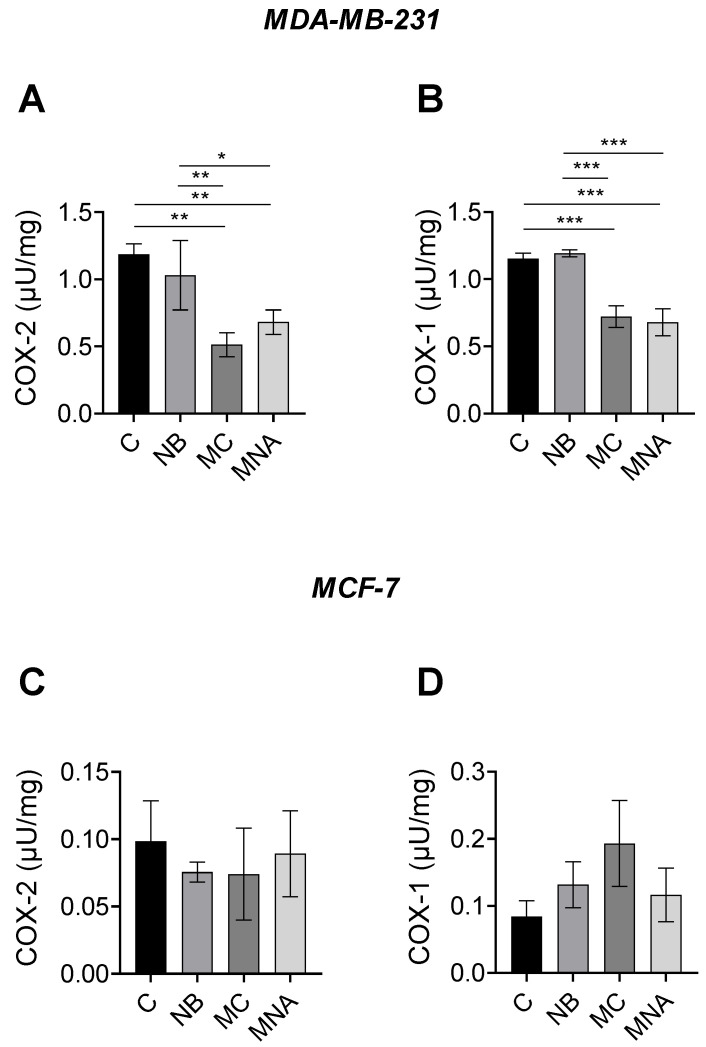
(**A**) COX-2 activity in MDA-MB-231 cells untreated (control, C) or treated with NB (35 µM), MC (25 µM) and MNA (35 µM). (**B**) COX-1 activity in MDA-MB-231 cells untreated (control, C) or treated with NB (35 µM), MC (25 µM), and MNA (35 µM). (**C**) COX-2 activity in MCF-7 cells untreated (control, C) or treated with NB (55 µM), MC (55 µM), and MNA (55 µM). (**D**) COX-1 activity in MDA-MB-231 cells untreated (control, C) or treated with NB (55 µM), MC (55 µM), and MNA (55 µM). In all cases, the cells were treated for 24 h, and all values are expressed as µU/mg protein. Data represent mean ± SEM, *n* = 3. * *p* ≤ 0.05, ** *p* ≤ 0.01, *** p≤ 0.001 (one-way ANOVA and the nonparametric Newman–Keuls multiple comparison test).

The results obtained from the predictive analyses and MTT assay prompted us to investigate the potential ability of NB, MC, and MNA to affect the activity of COX-1 and COX-2 in both MDA-MB-231 and MCF-7 cell lines. For this purpose, in the MDA-MB-231 cell line, NB, MC, and MNA were tested at 35, 25, and 35 µM, respectively, while in the MCF-7 cell line, all the three compounds were tested at 55 µM. As reported in Figure 9A, both MC and MNA significantly inhibited COX-2 activity compared to the control group in MDA-MB-231 cells. A similar trend was shown by NB, although to a much lesser extent. Comparable results were observed for COX-1 activity in MDA-MB-231 cells. Accordingly, except for NB, in MC- and MNA-treated cells, the activity of COX-1 resulted significantly decreased compared to the control group (Figure 9B). Conversely, in MCF-7 cells, all the three compounds did not significant affect the activity of COX-1 and COX-2 compared to control cells (Figure 9C,D).

In conclusion, our compounds, in particular MC, were found to be more active against COX-2 in the MDA-MB-231 cells, which are known to express a higher level of this isoform, confirming the results obtained through in silico studies.

### 2.5. Effects of NB and MC on Cell Cycle

**Figure 10:**
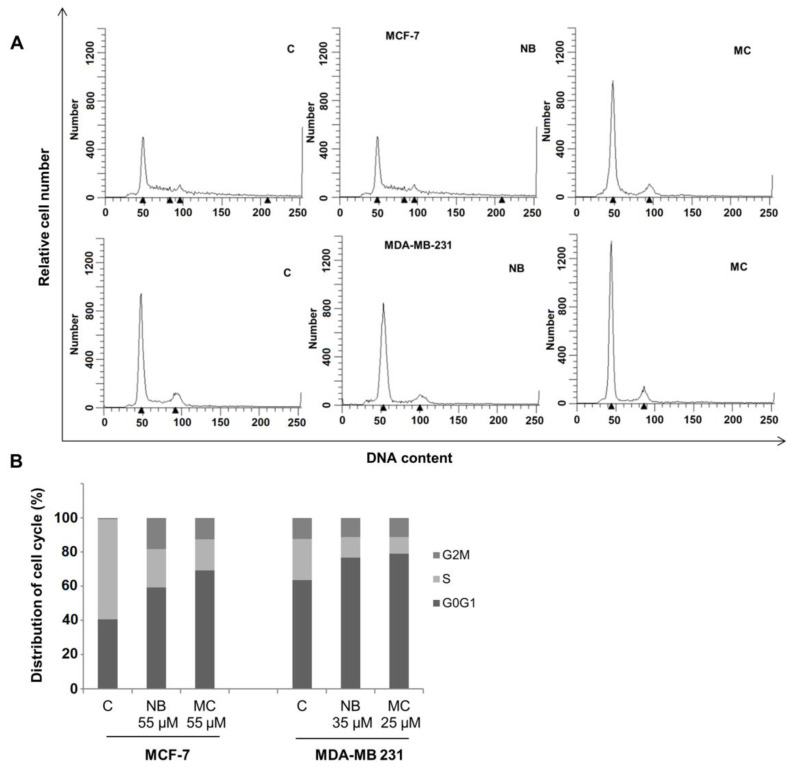
Flow cytometry analysis of the cycle profile of breast cancer cells. The proliferative data are expressed by the percentage of cells across the various phases of the cell cycle. (**A**) DNA content: DNA of individual cells; Relative cell number: distribution of cells across the cell cycle phases. MCF-7 treated with NB 55 µM and MC 35 µM, MDA-MB-231 treated with NB 55 µM and MC 25 µM, for 24 h. (**B**) Quantitative analysis of the fraction of gated cells at G0/G1, S and G2/M phases. The results are representative of three independent experiments. C = control.

Cell cycle perturbations induced by NB and MC were examined in MCF-7 and MDA-MB-231 cell lines. Tests were performed by flow cytometry analysis of DNA profiles after 24 h treatment, at concentrations for which a cell survival of 50% was observed. As shown in Figure 10A, NB and MC caused an increase of cell retained at the G0/G1 phase in MCF-7 of 59.36% and 69.25%, respectively, compared to 40.66% for the control. A reduction of 22.46% and 18.20% of cell population in the S-phase was observed for NB and MC, respectively. In MDA-MB-231 cells, an increase of 76.83% and 79.04% at the G0/G1 phase was observed for NB and MC, respectively (control was 63.52%), with a concomitant reduction of cell population in the S-phase of 11.91% and 9.85% in the two respective cases (Figure 10B). These results are in accordance with data reported in the literature for DOXO and TAM [45].

The results obtained after cytotoxicity and cell cycle assays confirm that both NB and MC determine a significant antiproliferative response on MCF-7 and MDA-MB 231 cancer cell lines. It is noteworthy that both compounds were more active, even at lower concentrations, against the more aggressive and invasive triple-negative MDA-MB-23 with respect to MCF-7 breast cancer cells [46].

### 2.6. Effects of NB and MC on the Expression of Cell Cycle Checkpoint Proteins

Loss of control of normal cell replication leads to aberrant proliferation, which is one of the main cause of cancer emergence [47]. Altered proteins involved in cell cycle regulation therefore represent molecular targets for anticancer chemotherapy. One of the species involved in the cell cycle regulation is represented by the p21 protein. The expression of such protein was observed to increase when the cell DNA is damaged and cells block at the G1 phase to avoid further DNA replication [48]. In fact, p21 acts as an inhibitor of the cyclin-associated kinases, which in turn control the activation–deactivation of cyclin-kinases complexes. In particular, the G1/S cell transition could be regulated by the cyclin D1/CDK4 complex [49]. Moreover, the expression of p21 could be modulated by p53, a tumor-suppressor protein that plays a crucial role in preventing cancer formation. An alteration of the p53 expression is usually correlated with cancer onset. Based on the results of cell cycle distribution, the expression of cell cycle checkpoint proteins was investigated in order to assess how NB and MC favor the cell cycle arrest.

**Figure 11:**
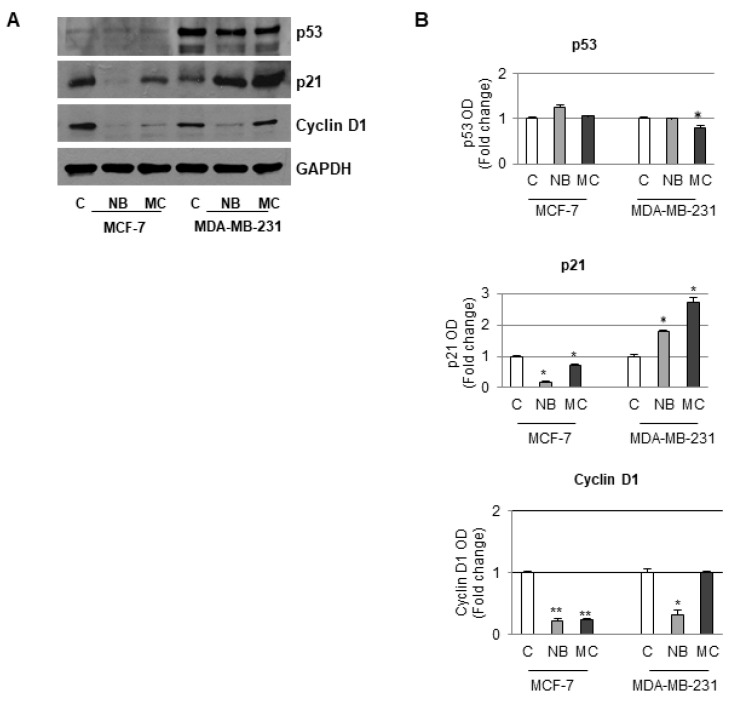
Immunoblotting analysis of p53, p21, and cyclin D1 protein levels in MCF-7 and MDA-MB-231 cells. (**A**) Sample untreated or treated for 24 h with 55 µM of NB or MC, 35 µM of NB, and 25 µM of MC, respectively. GAPDH was used as loading control. (**B**) The histograms represent the mean ± SD of two separate experiments in which band intensities were evaluated in terms of optical density arbitrary units (OD) and expressed as fold change versus control (C) * *p* ≤ 0.05. ** *p* ≤ 0.001.

Since in our experiments there was an increase in the number of cells in the G1 phase, the D1 cyclin, p21 and p53 expression was accordingly investigated on both MCF-7 and MDA-MB-231 cell lines. In particular, in MCF-7 cells containing a functional p53 protein, a reduction in the expression of both D1 cyclin and p21 with a concomitant increase of p53 after treatment with NB or MC (55 µM) was observed after Western blotting analysis. In the case of MDA-MB-231, which contains a mutated type of p53 that does not control p21 activity, treatment with NB (35 µM) did not cause a significant reduction in p53 expression even though an increase in p53-independent p21 expression, at concentration lower than that used in the former cell line, was observed. A comparable result was obtained after treatment with MC, even at a lower concentration (25 µM). Furthermore, in this cell line, the D1 cyclin expression was reduced after NB treatment, whereas treatment with MC did not produce any significant changes with respect to the control. Glyceraldehyde-3-phosphate dehydrogenase (GAPDH) was used as an internal control due to its important role in cancer cells in which many factors, including p53, intervene to modulate the expression [50]. However, based on previous experience, no correlation between the two proteins GAPDH and p53 expression was proven for several cancer models [51,52,53,54,55,56,57]. It is worth noting that in our experiments, even though equal amounts of proteins were loaded, no difference in GAPDH bands was observed between control and treated cells, suggesting that our compounds did not affect GAPDH expression. In addition, there was no effect of the compounds on the expression of p53, which is in turn among the factors regulating the expression of GAPDH (Figure 11).

Taken all together, these results suggest that poorly differentiated MDA-MB-231 cells, which are recognized as highly aggressive and invasive, are more sensitive to the action of both tested compounds. In conclusion, NB and MC might be able to circumvent p53 mutation, which is often associated with poor prognosis and therapeutic resistance in triple negative MDA-MB-231 cells.

## 3. Materials and Methods

### 3.1. Molecular Docking

Molecular docking of MNA, NB, and MC was carried out on the crystallographic structure of COX-2 (PDB code 5IKR) [43], and of COX-1 (PDB code 6Y3C). The molecular structures of the ligands were built by using the modeling software Avogadro [58]. Preliminary conversion of the structures from the PDB format was carried out by using the graphical interface AutoDock Tools 1.5.6 [59]. During the conversion, polar hydrogens were added for the crystallographic enzymes, and apolar hydrogens of MNA, NB, and MC were merged to the carbon atom they were attached to. Docking calculations were performed by using AutoDock Vina 1.1.2 [60], either exploring the search volume that included the protein structure or by performing a score-only assessment without any search in the case of re-docking of the crystallographic ligand in its known binding pose. In the former case, a very high exhaustiveness of search was used, eight time larger than the default value [61,62], facilitated by the relatively small number of active torsions around bond dihedral angles necessary to give full flexibility to the three ligands (four for NB and MNA, one for MC). The binding modes of the ligands were analyzed through visual inspection, and interactions energies and distances were quantified by using Molecular Operating Environment (MOE) 2018.01 (Chemical Computing Group ULC, Montreal, Canada). The Molecular Graphics System PyMOL [63] was used to visualize protein structure and ligand binding.

### 3.2. Cell Culture

Human breast cancer epithelial cell line MCF-7 (ER+ positive) and triple-negative human breast cancer cell line MDA-MB 231 (ER-, PR-, HER2- negative) were cultured in DMEM-F12 containing 10% Fetal Bovine Serum (FBS) (Life Technologies, Monza MB, Italy) at 37 °C with 5% CO_2_ flow. Human normal breast epithelial cell line MCF-10A was grown in DMEM-F12 medium containing 5% horse serum. Cell lines used were acquired from ATCC (American Type Culture Collection, Manassas, USA). They were authenticated, stored following manufacturer’s instructions, and used within 4 months after frozen aliquot resuscitations.

### 3.3. Compound Dilutions

NB (Merck, Darmstadt, Germany, cat. nr. N0020020, purity >98.5%); MNA (Merck, Darmstadt, Germany, cat. nr. CDS014591, purity > 98.5%); MC (synthesized in house, purity >99%, [34]), DOXO (Merck, Darmstadt, Germany, cat.nr. D1515, purity >98%), and TAM (Merck, Darmstadt, Germany, cat. nr. T5648, purity > 99%) were dissolved in DMSO to prepare 10 mM stock solutions and stored at −20 °C. Further dilutions were freshly made in cell culture medium.

### 3.4. Cell Viability Assays

Cell viability was determined with the 3-(4,5-dimethylthiazol-2-yl)-2,5-diphenyl tetrazolium (MTT, Merck, Darmstadt, Germany) assay. MCF-7 and MDA-MB-231 cells were grown in 96-well plates and exposed to treatments as indicated for 24 and 48 h in phenol red-free DMEM-F12 containing 5% charcoal-stripped FBS (cs-FBS). The MTT assay was performed as follows: 100 µL MTT stock solution in PBS (2 mg/mL) was added into each well and incubated at 37 °C for 2 h followed by media removal and solubilization in 100 µL DMSO. After shaking the plates for 15 min, the absorbance at 570 nm was measured in each well, including the blanks, in a Beckman Coulter Spectrophotometer (Midland, Canada).

### 3.5. COX Activity Assay

COX-1 and COX-2 activity were estimated using a Fluorometric Cyclooxygenase Activity Assay (Abcam, ab204699, Cambridge, UK) following the manufacturer’s instruction. 10^6^ MDA-MB-231 cells were seeded in DMEM-F12 complete medium and treated with NB (35 μM), MC (25 μM), or MNA (35 μM) for 24 h, while 10^6^ MCF-7 cells were seeded in DMEM-F12 complete medium and treated with NB (55 μM), MC (55 μM), or MNA (55 μM) for 24 h. After reaching a confluence of ~80%, cells were lysed in lysis buffer with protease inhibitor cocktail. Cell lysates were centrifuged at 12.000× *g* for 3 min at 4 °C and supernatants were collected for COX activity estimation. Fluorescence (λ_Ex/Em_ = 535/587 nm) was measured in a kinetic mode using a microplate reader and COX-1 and COX-2 activity were expressed as μU/mg.

### 3.6. Cell Cycle Analysis

Cell cycle perturbations were analyzed by propidium iodide DNA staining. Briefly, MCF-7 and MDA-MB-231 were treated with NB and MC at the reported doses for 24 h, then collected for cell cycle analysis. Cells were washed with PBS and fixed for 1 h in ice-cold 70% ethanol. The samples were then washed once with PBS and suspended in 1 ml of staining solution (10 mg/mL RNasi A, 10 mg/mL propidium iodide in PBS) and then incubated at room temperature in the dark for at least 30 min. Cell cycle profiles were obtained by using a FACScan flow cytometer (Broomfield, CO, USA) and data were analyzed using ModFit LT software (Becton Dickinson, NJ, USA). At least 2 × 10^4^ cells/sample were measured.

### 3.7. Immunoblotting Analysis

MCF-7 and MDA-MB-231 cells were grown up to 70–80% confluence and treated in 5% cs-FBS media before lysis in 500 µL of 50 mM Tris–HCl, 150 mM NaCl, 1% NP-40, 0.5% sodium deoxycholate, 2 mM sodium fluoride, 2 mM EDTA, 0.1% SDS, containing a mixture of protease inhibitors (aprotinin, phenylmethylsulfonyl fluoride, and sodium orthovanadate; Merck, Darmstadt, Germany) for total protein extraction. Equal amounts of proteins were resolved on 8% SDS-polyacrylamide gel, transferred to a nitrocellulose membrane and probed with cyclin D1, p53, p21^Cip/WAF1^ and GAPDH specific antibodies (Santa Cruz Biotechnology, CA, USA). The antigen-antibody complex was detected by incubation of the membranes with peroxidase-coupled goat anti-mouse or goat anti-rabbit antibodies and revealed using the ECL System (Amersham Pharmacia, Buckinghamshire, UK).

### 3.8. Statistical Analysis

Data were analyzed for statistical significance using two-tailed Student’s t-test (GraphPad-Prism4 software). A one-way ANOVA and the nonparametric Newman–Keuls multiple comparison test (for post-ANOVA comparisons) was used for COX activity analysis. These data were expressed as means ± SEM and * *p* ≤ 0.05, ** *p* ≤ 0.01, *** *p* ≤ 0.001 were considered statistically significant. For COX activity estimation, the statistical analysis was carried out using Graphpad-Prism5 software.

## 4. Conclusions

The direct COX-2 inhibition by NB and its tricyclic analog MC has not yet been studied in vivo because of their rapid metabolic transformation into MNA, a well-known inhibitor of such enzyme. The molecular docking experiments performed suggest the ability of these prodrugs to interact with the COX-2 binding site in a similar way to their common active metabolite, thus confirming the hypothesis of some intrinsic anti-enzymatic activity, which does not exclude therapeutic properties other than anti-inflammatory. NB and MC were then envisaged as antiproliferative agents to be included in specific formulations. NB and MC were found to be able to inhibit cell growth in both estrogen positive MCF-7 and triple negative MDA-MB-231 cancer cells, leading to cell cycle arrest in the G1 phase. The same compounds did not exert any significant cytotoxic effect on non-transformed cells. The combination of the direct antiproliferative action with the possible COX-2 inhibition exerted by the molecules may be useful for the design of innovative depot anticancer formulations.

## Figures and Tables

**Table 1:** Distance between the crystallographic ligand (MEF) and docking poses of the ligands, calculated among the geometric centers of the selected region.

	All Non-Hydrogen Atoms (Å)	Closest Aromatic Ring (Å)
**MNA**	1.53	0.45
**MC**	1.80	1.15
**NB**	2.84	0.90
**NAP**	1.54	0.45
**DIC**	0.42	0.65

**Table 2:** IC_50_ of NB and MC for MCF-7 and MDA-MB-231 cell lines. IC_50_ is defined as the drug concentration causing a 50% decrease in cell population after 24 h culture. CI = confidence interval.

Compound	MCF-7		MDA-MB-231	
	IC_50_ (µmol/L)	95% CI	IC_50_ (µmol/L)	95% CI
NB	55.03	51.99–58.25	32.55	29.36–36.11
MC	55.03	52.03–58.36	24.80	18.11–33.97

## Sample Availability

Samples of the compounds are not available from the authors.
